# Development and application of a TaqMan-probe-based multiplex real-time PCR assay for simultaneous detection of porcine circovirus 2, 3, and 4 in Guangdong province of China

**DOI:** 10.3389/fvets.2024.1353439

**Published:** 2024-04-26

**Authors:** Pian Zhang, Zhaowen Ren, Xiaopeng Gao, Mengpo Zhao, Yanyun Wang, Jing Chen, Gang Wang, Hua Xiang, Rujian Cai, Shengjun Luo, Xiaohu Wang

**Affiliations:** ^1^Key Laboratory of Livestock Disease Prevention of Guangdong Province, Key Laboratory for Prevention and Control of Avian Influenza and Other Major Poultry Diseases, Ministry of Agriculture and Rural Affairs, Guangdong Provincial Observation and Research Station for Animal Disease, Institute of Animal Health, Guangdong Academy of Agricultural Sciences, Guangzhou, China; ^2^College of Veterinary Medicine, South China Agricultural University, Guangzhou, China

**Keywords:** porcine circoviruses, multiplex real-time PCR, prevalence, genotype, phylogenetic analysis

## Abstract

Porcine circoviruses disease (PCVD), caused by porcine circovirus (PCVs), is an important swine disease characterized by porcine dermatitis, nephrotic syndrome and reproductive disorders in sows. However, diseases caused by PCV2, PCV3, or PCV4 are difficult to distinguish, so a simple, rapid, accurate and high-throughput diagnostic and identification method is urgently needed to differentiate these three types. In this study, specific primers and probes were designed based on the conserved region sequences of the Rep gene of PCV2, and the Cap gene of PCV3 and PCV4. A multiplex qPCR assay was developed and optimized that the limit of detection concentration could reach as low as 3.8 copies/μL, with all correlation coefficients (R^2^) exceeding 0.999. Furthermore, the method showed no cross-reaction with other crucial porcine viral pathogens, and both intra-repeatability and inter-reproducibility coefficients of variation were below 2%. The assay was applied to the detection of 738 pig samples collected from 2020 to 2021 in Guangdong Province, China. This revealed positive infection rates of 65.18% for PCV2, 29.27% for PCV3, and 0% for PCV4, with a PCV2/PCV3 co-infection rate of 23.17%. Subsequently, complete genome sequences of 17 PCV2 and 4 PCV3 strains were obtained from the above positive samples and pre-preserved positive circovirus samples. Nucleotide sequence analysis revealed that the 17 PCV2 strains shared 96.7–100% complete nucleotide identity, with 6 strains being PCV2b and 11 strains being PCV2d; the 4 PCV3 strains shared 98.9–99.4% complete nucleotide identity, with 2 strains being PCV3a-1 and 2 strains being PCV3b. This research provides a reliable tool for rapid PCVs identification and detection. Molecular epidemiological investigation of PCVs in pigs in Guangdong Province will help us to understand PCV2 and PCV3 epidemiological characteristics and evolutionary trends.

## 1 Introduction

Porcine circoviruses (PCVs) are circular, non-enveloped, single-stranded DNA viruses that are prevalent worldwide and pose a serious challenge to pig industry development (1). Circoviruses have the smallest genome length of all known animal viruses, ranging from 1758 to 2000 nt and containing two major open reading frames (ORFs). The ORF1 gene is located on the genome sense strand and encodes the replication-associated protein (Rep), while the ORF2 gene is located in the opposite orientation and encodes the capsid protein (Cap), which determines the antigenic characteristics of circoviruses (2). Currently, four species of PCVs have been identified, including PCV1, PCV2, PCV3, and PCV4 (3–5). PCV1 is generally considered non-pathogenic and is widespread in pigs; it also plays as a source of contamination of cell lines (6). PCV2 is considered a causative agent of porcine circovirus diseases (7). Based on phylogenetic analysis of the genome and ORF2 sequences, PCV2 can be divided into 8 subtypes (PCV2a-PCV2h) (8). Among them, PCV2a, PCV2b, and PCV2d are transmitted in pig populations worldwide, whereas PCV2c is currently isolated only from samples from Brazil and Denmark, and PCV2e is mainly distributed in the United States and Mexico (9, 10). PCV3 was initially detected in pigs with porcine dermatitis and nephropathy syndrome (PDNS) in the United States using high-throughput sequencing in 2016 (2, 11). It is not yet apparent how PCV3 contributes to illness pathogenesis or whether disease-related damage can occur. In addition, PCV3 has been found in a variety of animals, including wild boar and farmed pigs (12, 13). Currently, there are several PCV3 genotyping methods, for example, based on the consistent differences in the amino acid positions 24 and 27 of the capsid protein, PCV3 is classified into three genotypes (PCV3a, PCV3b, and PCV3c). There is no apparent correlation between PCV3 subtypes and their source strains (8, 14, 15). Finally, PCV4 was initially detected in Hunan Provence, China, in 2019, and has been reported in recent years in Guangxi Province, Henan Province and Sichuan Province in China (5, 16–18). It has been suspected to be associated with severe clinical conditions, such as PDNS, respiratory signs, and enteric manifestations (16, 19).

PCVs infection in pigs can be either a single genotype or a co-infection with multiple genotypes. Due to their similar clinical symptoms, molecular biology methods are usually required for differential diagnosis in clinical settings (20). Currently, commonly used PCVs detection methods include enzyme-linked immunosorbent assay (2, 21), and conventional PCR. Although these methods can detect PCV, they are time-consuming, cumbersome, and unable to simultaneously identify multiple pathogens, so cannot fully meet practical production needs. Therefore, in this study, a TaqMan triple real-time PCR assay for rapid differential detection of PCV2, PCV3 and PCV4 was established and also applied in an epidemiological PCVs investigation in Guangdong Province from 2020 to 2021. Complete genome sequence amplification and phylogenetic analysis of PCV2 and PCV3 were performed on the 738 samples collected from pig farms in Guangdong Province from 2020 to 2021 and PCV-positive samples kept in our laboratory from 2015 to 2021. Our aim was to provide detection tools and relevant references for clinical diagnosis and epidemiologic investigation of PCVs.

## 2 Materials and methods

### 2.1 Primers and probes

A total of 282 PCV complete genomes (175 PCV2, 82 PCV3, and 25 PCV4) were downloaded from the GenBank database. Using MEGA-X software, a multiple sequence alignment was performed to select specific conserved regions. The conserved region of Rep was chosen for designing the primer and probe for PCV2, and the Cap conserved region was chosen for PCV3 and PCV4. Specific primers and probes for each type were designed at the conserved sequences using primer 6.0 software, and qPCR amplification fragment sizes were as follows: PCV2 (116 bp), PCV3 (137 bp) and PCV4 (75 bp). All primer and probe sequences synthesized by Beijing Tsingke Biotech Co., Ltd. (Beijing, China) are shown in Table 1.

**Table 1:** Primers and probes used in this study.

Primers/probes	Sequences (5′ − 3′)	Positions	Genes	Amplicons
PCV2-F	ACGAGCGCAAGAAAATACGG	130–149	Rep	116 bp
PCV2-R	CACAAAATTAGCGAACCCCT	226–245		
PCV2-P	FAM-CCTCATTACCYTCCTCG CCAAC-BHQ1	183–204		
PCV3-F	GCCGTAGAAGTCTGTCATTCC	1,375–1,395	Cap	137 bp
PCV3-R	CTCACCCAGGACAAAGCC	1,494–1,511		
PCV3-P	HEX-AACGGTGGGGTCATATGTGTTGAGCCATGG-BHQ1	1,441–1,470		
PCV4-F	GATCCACCATTGGTTTCTTTTGTTG	1,191–1,215	Cap	75 bp
PCV4-R	AAACCCCAGGACCCATCCG	1,247–1,265		
PCV4-P	Cy5-ACCCACACCCTCCACTTCCAGCCT-BHQ3	1,218–1,241		

*Positions were determined according to PCV2 YiY-1-1 strain (KU311020), PCV3 HeNYS-3 strain (MH184541), and PCV4 HNU-AHG1-2019 strain (MK986820), respectively.

### 2.2 Virus and clinical samples

All viruses used for specificity validation were obtained from commercial vaccines purchased from Jilin Zhengye Biological Products Co., Ltd. (Jilin, China), including Porcine Reproductive and Respiratory Syndrome virus (PRRSV, HuN4-F112), Pseudorabies virus (PRV, Bartha-K61), Porcine Transmissible Gastroenteritis virus (PTGV), Porcine Epidemic Diarrhea virus (PEDV), Porcine Rotavirus (PoRV), and Classical Swine Fever virus (CSFV).

During 2020–2021, a total of 738 samples were collected from swine farms in Guangdong province and preserved in our laboratory, including 326 nasopharyngeal swab samples and 412 blood samples (Table 2). Molecular epidemiological investigation of the above samples was conducted using the established multiplex qPCR assay established in this study. Furthermore, complete genome amplification and genetic evolutionary analysis were also performed on the positive samples detected in 2020–2021 as described above and PCV-positive samples kept in our laboratory from 2015 to 2021.

**Table 2:** Total number of pig samples collected in Guangdong Province from 2020 to 2021.

Region	Number of pig farms	Number of samples
Pearl River Delta	25	246
Northern Guangdong	16	187
Eastern Guangdong	4	113
Western Guangdong	15	192
Total	60	738

### 2.3 DNA/RNA extraction and reverse transcription

RNA/DNA was extracted from samples or vaccines using a RaPure Viral RNA/DNA Kit (Magen, Guangzhou, China) according to the manufacturer’s instructions. First-strand cDNA was synthesized using extracted viral RNA and a RevertAid First Strand cDNA Synthesis Kit (Thermo Scientific, USA). DNA and cDNA products were stored at −80°C.

### 2.4 Recombinant plasmid construction

To construct the recombinant standard plasmid, the multiplex qPCR assay amplification products (PCV2-PCV3-PCV4) were connected together as a standard sequence for synthesis (Tsingke, Beijing, China). The synthesized sequence was cloned into the pUC57 vector and transformed into DH5α competent cells. Then, the bacterial cultures were shaken and grown for 16 h at 37°C. The recombinant plasmid was purified using a GeneJET Plasmid Miniprep Kit (Thermo Scientific, USA) and the concentration was determined using Qubit 4.0 (Thermo Scientific, USA). The final plasmid obtained was named PUC57-PCVs. The plasmid concentration was converted into a copy number using the following formula:

$$y\left(\mathrm{copies}/\mathrm{\mu L}\right)=\frac{\left(6.02\times 10^{23}\right)\times \left(x\;\mathrm{ng}/\mathrm{\mu L}\times 10^{-9}\right)}{DNA\;\mathrm{length}\times 660}$$

### 2.5 Multiplex qPCR assay optimization

The multiplex qPCR conditions were optimized, including annealing temperature, primer and probe concentrations. The reaction system was 20 μL, including 10 μL of 2 × AceQ Universal U+ Probe Master Mix V2 (Vazyme, Nanjing, China), 2 μL of templates, different final concentrations of primers, probes, and nuclease-free water. The Optimization of the reaction conditions was performed utilizing a matrix approach with annealing temperatures ranging from 50°C to 60°C; final primer concentrations spanning from 100 nM to 500 nM in nine incremental dilutions; and final probe concentrations set at 50, 100, 150, and 200 nM, respectively. Amplification conditions were 37°C for 2 min, pre-denaturation at 95°C for 300 s, followed by 40 cycles of 95°C for 10 s, and 60°C for 30 s. The qPCR instrument fluorescence channels were configured as follows: channel 1, FAM; channel 2, HEX; and channel 3, Cy5. Fluorescence signals were collected by a LightCycler® 96 Instrument (Roche Applied Science, Penzberg, Germany). Standard plasmids were diluted from 3.8 × 10^6^ copies/μL to 3.8 × 10^2^ copies/μL as amplification templates, and the system was optimized by generating the lowest threshold cycle (Ct) and the highest cycle increase (ΔRn) for each specific fluorescent signal.

### 2.6 Standard curve construction

Based on the optimal reaction system, the standard plasmid was diluted 10-fold from 3.8 × 10^6^ copies/μL to 3.8 × 10^2^ copies/μL, and multiplex qPCR was performed with five concentrations of the standard plasmid as templates. The standard curve was plotted using Prism software.

### 2.7 Specificity, sensitivity, repeatability assay

To assess the specificity of the multiplex qPCR assay, the PUC57-PCVs standard plasmid was used as the positive control and ddH_2_O as the negative control, DNA/cDNA of PRRSV, PEDV, CSFV, PTGV, PoRV and PRV extracted from the vaccine strains were used as templates for the qPCR assay detection. To determine the sensitivity, the standard plasmid was diluted 10-fold from 3.8 × 10^6^ copies/μL to 3.8 × 10^0^ copies/μL, qPCR amplification was performed in an optimal reaction system, and three replicates of each concentration were performed to confirm the detection limit of this multiplex qPCR assay. Intra-assay and inter-assay reproducibility was assessed using standard plasmids (3.8 × 10^6^ copies/μL to 3.8 × 10^2^ copies/μL) as templates. For intra-assay reproducibility, three parallel replicates of each dilution were performed daily under the same conditions, and for inter-assay reproducibility, each dilution was tested by six independent experiments performed by two operators on different days according to the MIQE guidelines (22). Coefficients of variation of the Ct values were calculated based on the intra-assay or inter-assay results.

### 2.8 Clinical sample detection

From 2020 to 2021, 738 clinical samples, including 326 nasopharyngeal swabs and 412 blood samples, were collected from swine farms in Guangdong Province, China. These samples were subsequently analyzed using an established multiplex qPCR assay to determine infection rates.

### 2.9 Complete genome amplification and sequencing

PCV2 (23) and PCV3 (20) complete genomes were amplified using the primers described previously. The reaction system was 50 μL, containing 25 μL of 2 × Platinum™ II Taq Hot-Start DNA Polymerase (Thermo Scientific, USA), 2 μL of template DNA, 200 nM primers, and the remaining was added to ddH_2_O. The PCR reaction for PCV2 was executed by pre-denaturation at 94°C for 2 min, followed by 35 cycles of 94°C for 15 s, 56°C for 15 s, and 68°C for 20 s. PCV3 amplification conditions were similar to those of PCV2, except that the PCV3 primer set was annealed at 57°C. The purified PCR products were cloned using a pMD18-T vector cloning kit (Takara, Dalian, China), and propagated in DH5α competent cells (Takara, Dalian, China) according to the manufacturer’s instructions. Positive clones were sequenced by Beijing Tsingke Biotech Co., Ltd. The obtained complete genome sequences were edited and assembled using DNAstar V7.1 software.

### 2.10 Sequence analysis

A total of 17 PCV2 and 4 PCV3 complete genomes were obtained in this study (Supplementary Table 1) and uploaded to NCBI GenBank with accession numbers MZ667312 (PCV2), MZ667314-MZ667329 (PCV2) and MZ667331-MZ667334 (PCV3). The complete PCV3 coding sequence (ORF1 + ORF2) was analyzed. Non-coding regions were deleted and the coding sequence was split into two parts, ORF1 (Rep) and ORF2 (Cap). Because ORF2 was in the opposite orientation, respective ORFs were downloaded and spliced separately. PCV3 subtypes were proposed by Li et al. (24). The PCV2 strain ORF2 sequence and the complete PCV3 coding sequence were aligned with the relevant reference sequences in GenBank using the ClustalW function of the Molecular Evolutionary Genetics Analysis software (MEGA, version 11.0), respectively. Phylogenetic trees were generated by the neighbor-joining (NJ) method in MEGA 11.0 with a p-distance model, and a bootstrap of 1,000 replicates.

## 3 Results

### 3.1 Optimization of qPCR reaction conditions

Annealing temperatures of 50, 55, and 60°C, primer final concentrations of 100–500 nM and probe final concentrations of 100–250 nM were selected for multiplex qPCR system optimization. Based on the optimization results, the annealing temperature was determined to be 60°C (Table 3), final primer concentrations for PCV2, PCV3, and PCV4 were 300, 350 and 200 nM, respectively (Supplementary Tables 2–4), and final concentrations of all three probes were 100 nM (Supplementary Table 5). Therefore, the final amplification system was 10 μL of 2× AceQ Universal U+ Probe Master Mix V2, 300 nM PCV2 primer, 350 nM PCV3 primer, and 200 nM PCV4 primer, each with 100 nM for each probe, 2 μL of template, and ddH_2_O was added to a final volume of 20 μL. Reaction conditions were 37°C for 2 min, pre-denaturation at 95°C for 300 s, followed by 40 cycles of 95°C for 10 s, and 60°C for 30 s.

**Table 3:** Optimization of annealing temperature of multiplex qPCR assay.

Annealing temperature	Template	Copy number (copies/μL)					Amplification efficiency (%)
		3.80 × 10^6^	3.80 × 10^5^	3.80 × 10^4^	3.80 × 10^3^	3.80 × 10^2^	
50°C	PCV2	16.38	20.53	24.26	28.05	30.49	91.39
	PCV3	15.77	19.87	23.37	27.24	29.65	92.60
	PCV4	13.67	17.94	21.66	25.08	27.72	92.21
55°C	PCV2	16.38	20.53	24.26	28.05	30.49	91.39
	PCV3	15.77	19.87	23.37	27.24	29.65	92.60
	PCV4	13.67	17.94	21.66	25.08	27.72	92.21
60°C	PCV2	16.88	21.64	24.45	28.53	30.57	94.10
	PCV3	16.09	20.96	23.62	27.56	29.56	98.68
	PCV4	14.27	18.83	21.75	25.56	28.11	95.26

### 3.2 Multiplex qPCR assay standard curve

**Figure 1:**
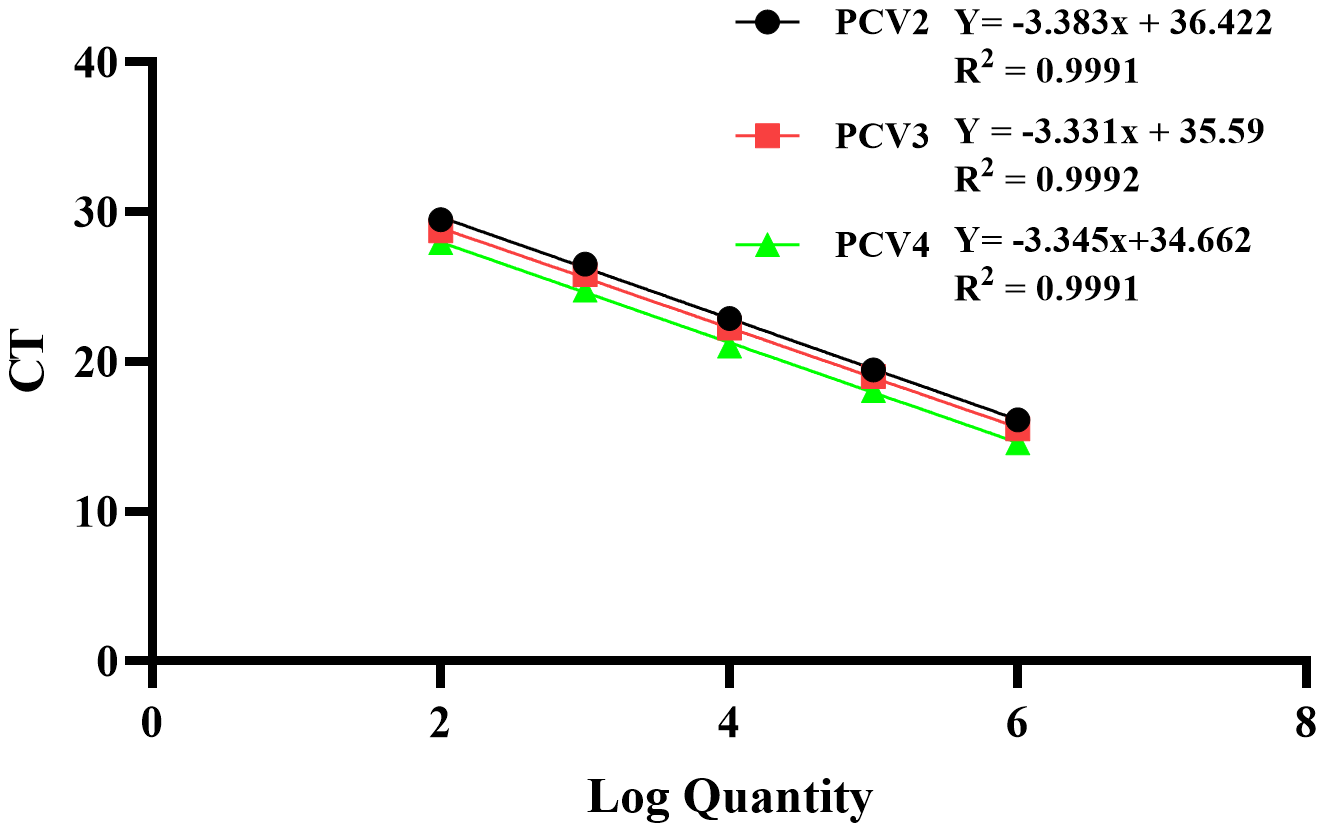
Standard curves of multiplex qPCR assay. Serial 10-fold dilutions of the standard plasmid were generated at final concentrations of 3.8 × 10^6^–3.8 × 10^2^ copies/μL. Data are from three independent studies. The threshold cycles (Ct) from the multiplex qPCR assay are plotted against the log numbers of the standards. Mean ± standard deviations are shown for each individual study.

The recombinant standard plasmid was diluted to 3.8 × 10^6^ to 3.8 × 10^2^ copies/μL in a 10-fold gradient, and multiplex qPCR assay was performed according to the optimal reaction system and procedure. The standard curve was established with the obtained Ct values as the *y* axis coordinates and the logarithm of the plasmid concentration as the *x* axis coordinates. All standard curves had good correlation coefficients and amplification efficiency. The slopes of PCV2, PCV3 and PCV4 were −3.383, −3.331 and −3.345, respectively; the correlation coefficient (R^2^) and amplification efficiency (Eff%) of the equation were as follows: PCV2 (*R*^2^ = 0.9991, Eff% = 97.51), PCV3 (*R*^2^ = 0.9992, Eff% = 99.62), PCV4 (*R*^2^ = 0.9991, Eff% = 99.05) (Figure 1).

### 3.3 Multiplex qPCR assay specificity

**Figure 2:**
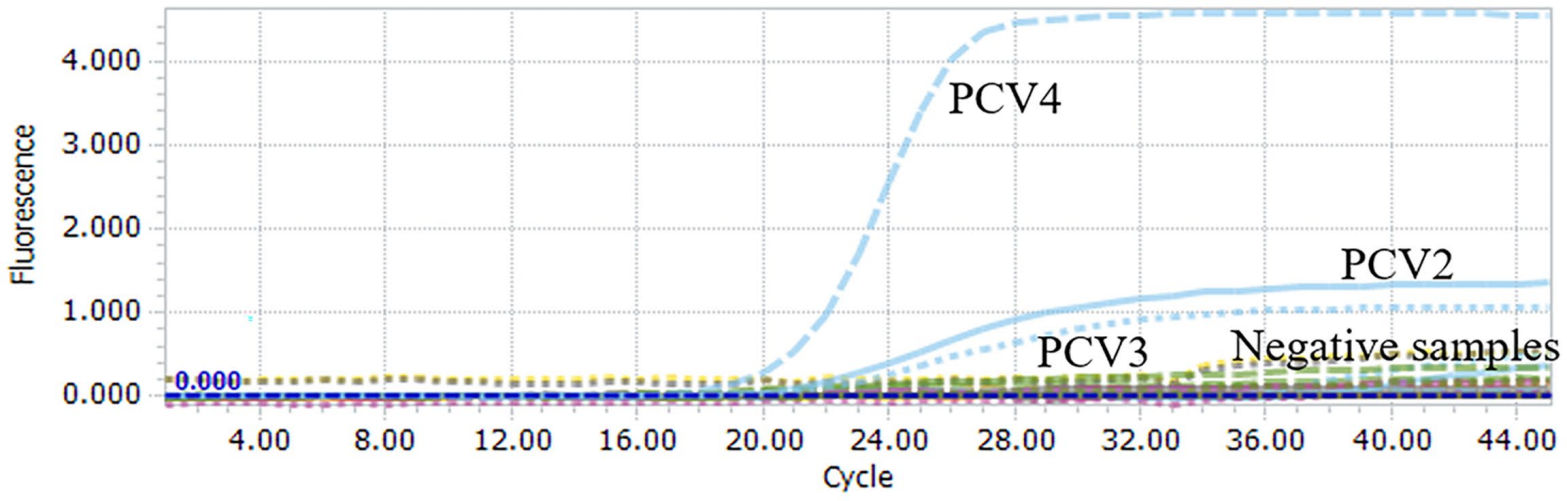
Specificity analysis of multiplex qPCR assay. When PUC57-PCVs plasmid was used, FAM, HEX and Cy5 fluorescent signals were specifically detected, respectively. No specific fluorescence signal was obtained when testing other viruses (PRRSV, PRV, PTGV, PEDV, PoRV, CSFV) and negative controls.

To assess the specificity of the multiplex qPCR, the recombinant standard plasmid PUC57-PCVs was used as a positive control. DNA/cDNA extracted from PRRSV, PEDV, CSFV, PTGV, PoRV, and PRV vaccines were used as templates, and ddH_2_O was used as a negative control. qPCR was performed using an optimal reaction system. Specific fluorescent signals for FAM, HEX and Cy5 were detected only when the PUC57-PCVs standard plasmid was used as a template. However, DNA and cDNA of other pathogens such as PRRSV, PEDV CSFV, and PRV were not amplified (Figure 2). These results support high specificity of the multiplex qPCR assay.

### 3.4 Multiplex qPCR assay sensitivity

**Figure 3:**
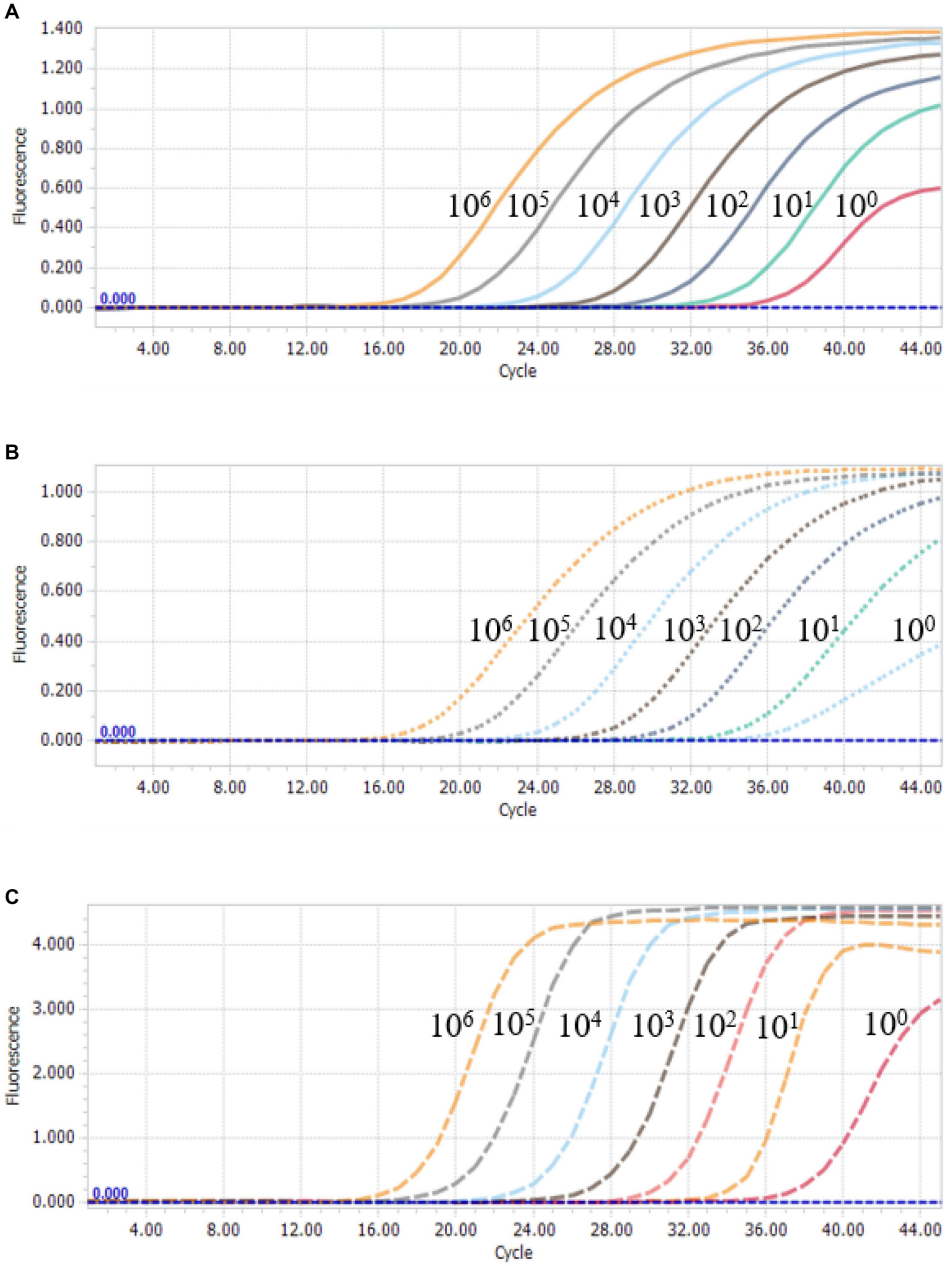
Sensitivity analysis of multiplex qPCR assay. Serial 10-fold serial dilutions of the standard plasmid were generated at final concentrations of 3.8 × 10^6^–3.8 × 10^0^ copies/μL. The multiplex qPCR assay has the detection limit at 3.8 × 10^0^ copies/μL for each species of PCVs. **(A)** Sensitivity analysis of PCV2. **(B)** Sensitivity analysis of PCV3. **(C)** Sensitivity analysis of PCV4.

To further assess the sensitivity of the multiplex qPCR assay, multiplex qPCR was performed using standard plasmids with concentrations ranging from 3.8 × 10^6^ to 3.8 × 10^0^ copies/μL. The results show that the detection limits of the assay were 3.8 × 10^0^ copies/μL for PCV2, PCV3 and PCV4 (Figure 3), suggesting that the multiplex qPCR assay has a good sensitivity.

### 3.5 Multiplex qPCR assay repeatability

To assess the reproducibility of the multiplex qPCR assay, standard plasmids with concentrations ranging from 3.8 × 10^6^ to 3.8 × 10^2^ copies/μL were tested, respectively. Intra- and inter-assay coefficients of variation (CV) of Ct values for the multiplex qPCR assay ranged from 0.10 to 0.77% and 0.47 to 1.45%, respectively (Table 4). These results indicated that the multiplex qPCR assay established in this study has good reproducibility and reliability.

**Table 4:** Repeatability and reproducibility analyses of multiplex qPCR assay.

Template	Copy number (copies/μL)	Intra reproductivity		Inter reproductivity	
		Mean ± SD	CV (%)	Mean ± SD	CV (%)
PCV2	3.80 × 10^6^	16.70 ± 0.11	0.69	16.35 ± 0.18	1.11
	3.80 × 10^5^	19.83 ± 0.11	0.54	19.72 ± 0.20	0.99
	3.80 × 10^4^	23.30 ± 0.02	0.11	23.16 ± 0.25	1.10
	3.80 × 10^3^	25.96 ± 0.06	0.23	25.69 ± 0.30	1.17
	3.80 × 10^2^	29.62 ± 0.12	0.41	29.31 ± 0.24	0.82
PCV3	3.80 × 10^6^	16.98 ± 0.13	0.77	16.14 ± 0.11	0.68
	3.80 × 10^5^	19.83 ± 0.06	0.30	20.03 ± 0.15	0.75
	3.80 × 10^4^	23.55 ± 0.04	0.17	23.39 ± 0.34	1.45
	3.80 × 10^3^	26.04 ± 0.05	0.19	25.67 ± 0.27	1.05
	3.80 × 10^2^	29.53 ± 0.12	0.41	29.70 ± 0.31	1.04
PCV4	3.80 × 10^6^	15.07 ± 0.11	0.73	14.82 ± 0.07	0.47
	3.80 × 10^5^	17.79 ± 0.13	0.73	17.80 ± 0.09	0.51
	3.80 × 10^4^	20.61 ± 0.02	0.10	20.72 ± 0.21	1.01
	3.80 × 10^3^	23.36 ± 0.08	0.34	23.10 ± 0.15	0.65
	3.80 × 10^2^	27.46 ± 0.14	0.51	26.37 ± 0.35	1.33

### 3.6 Clinical sample detection

738 samples collected from different swine farms in Guangdong Province during 2020–2021 were tested using the method established in this study. Among the 738 samples, PCV2 infection rate was 65.18% (481/738), PCV3 infection rate was 29.27% (216/738), and PCV2/PCV3 co-infection rate was 23.17% (171/738) (Table 5). In the Pearl River Delta, western Guangdong, eastern Guangdong and northern Guangdong, PCV2 infection rate ranged from 50.44 to 71.95%, PCV3 infection rate ranged from 25.0 to 33.74%, and PCV2 and PCV3 co-infection rate ranged from 16.15 to 29.27%. For nasopharyngeal swabs, PCV2 infection rate was 70.25% (229/326), PCV3 infection rate was 25.77% (84/326), and PCV2/PCV3 co-infection rate was 21.17% (69/326). For blood samples, infection rate for PCV2 was 61.17% (252/412), 32.04% for PCV3 (132/412), and for PCV2/PCV3 co-infection rate was 24.76% (102/412) (Table 6). PCV4 was not detected in the above samples.

**Table 5:** Summary of PCVs detection in 738 pig samples.

Region	PCV2 positive/total (%)	PCV3 positive/total (%)	PCV4 positive/total (%)	PCV2 + PCV3 positive/total (%)
Pearl River Delta	177/246 (71.95%)	83/246 (33.74%)	0//246 (0%)	72/246 (29.27%)
Northern Guangdong	124/187 (66.31%)	53/187 (28.34%)	0/187 (0%)	46/187 (24.6%)
Eastern Guangdong	57/113 (50.44%)	32/113 (28.32%)	0/113 (0%)	22/113 (19.47%)
Western Guangdong	123/192 (64.06%)	48/192 (25.0%)	0/192 (0%)	31/192 (16.15%)
Total	481/738 (65.18%)	216/738 (29.27%)	0/192 (0%)	171/738 (23.17%)

**Table 6:** Detection results of PCVs in different types of pig samples.

Sample type	PCV2 positive/total (%)	PCV3 positive/total (%)	PCV4 positive/total (%)	PCV2 + PCV3 positive/total (%)
Nasopharyngeal swab	229/326 (70.25%)	84/326 (25.77%)	0/326 (0%)	69/326 (21.17%)
Blood	252/412 (61.17%)	132/412 (32.04%)	0/412 (0%)	102/412 (24.76%)

### 3.7 PCV2 genetic characteristics

**Figure 4:**
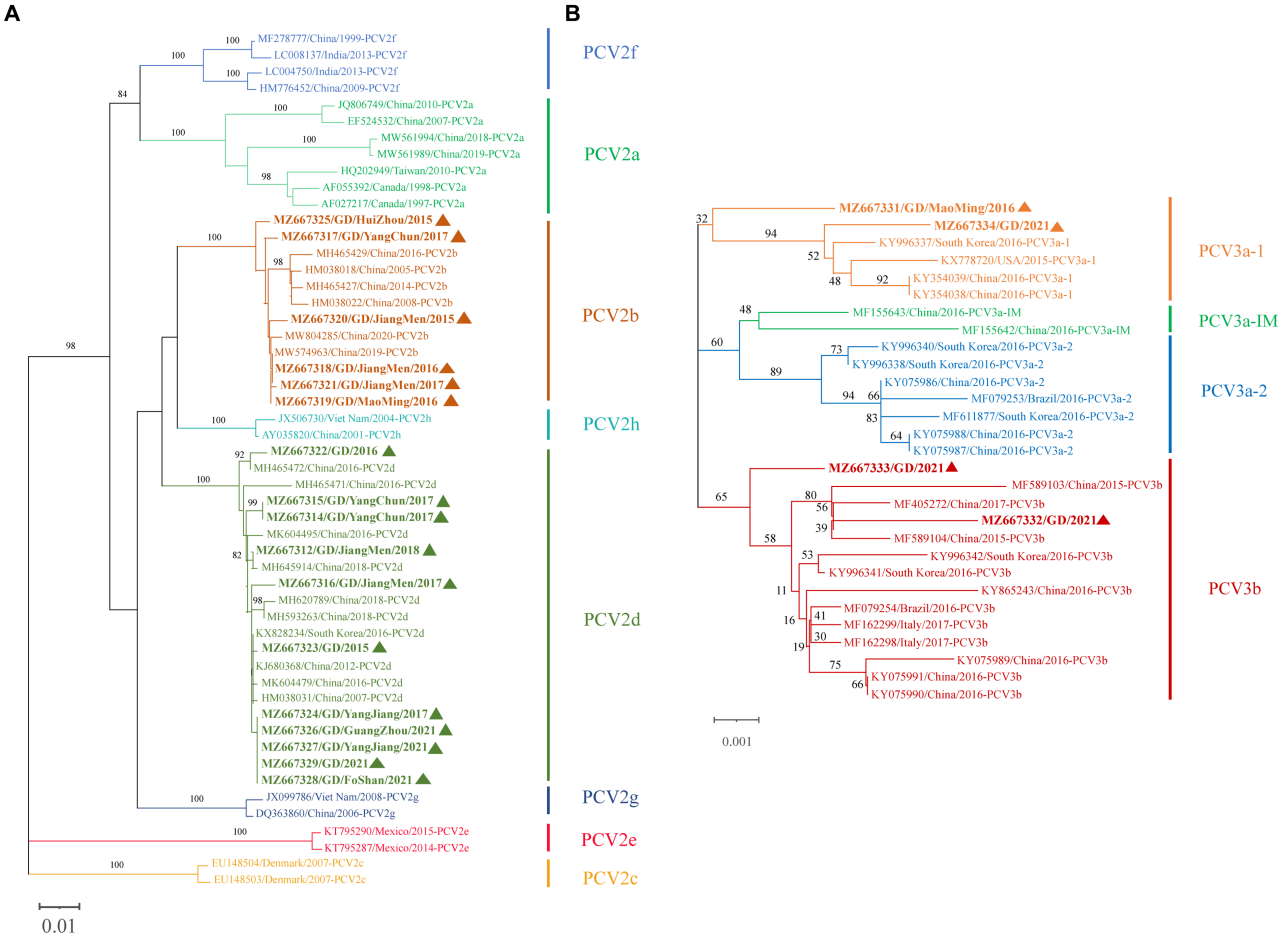
Phylogenetic tree based on ORF2 gene of PCV2 and the complete coding sequence (ORF1 + ORF2) of PCV3. The NJ tree was constructed using the p-distance model and bootstrapped at 1000 replicates. **(A)** PCV2. **(B)** PCV3. PCV3 isoforms were proposed by Li et al. (24). The sequences of PCV2 and PCV3 obtained in this study are marked with triangles. The different genotypes are represented by different colors as indicated in the figures.

We attempted Complete genome amplification from the above samples and PCV-positive samples kept in our laboratory from 2015 to 2021. A total of 17 PCV2 complete genomes were obtained, each of which was 1767 nt in size. A phylogenetic tree was constructed based on the ORF2 sequences of the 17 PCV2 strains and 35 PCV2 reference sequences (PCV2a-h) (Figure 4A). 17 PCV2 sequences we obtained could be classified into PCV2b (6 strains) and PCV2d (11 strains) subtypes. Nucleotide sequence analysis revealed that the 17 PCV2 strains shared 96.7–100% complete nucleotide identity and 94.0–100% ORF2 identity. Compared with the 35 PCV2 reference strains, the nucleotide identity of complete nucleotide and ORF2 varied 92.0 to 99.9% and 82.9 to 100%.

### 3.8 PCV3 genetic characteristics

Similarly, we successfully obtained 4 PCV3 complete genomes, all of which was 2000 nt in size, which were submitted to NCBI GenBank. A phylogenetic tree was constructed based on the coding gene sequences (ORF1 + ORF2) of the 4 PCV3 strains and the reference sequences of 25 PCV3 strains (Figure 4B). Among the 4 PCV3 strains we obtained, two belonged to PCV3b and two belonged to PCV3a-1, which had different clustering levels. Nucleotide sequence analysis showed that the 4 PCV3 strains shared 98.9–99.4% complete nucleotide identity and 98.3–99.2% ORF2 identity. Compared with the 25 PCV3 reference strains, the nucleotide identity of complete nucleotide and ORF2 varied 98.8 to 99.7% and 97.8 to 99.7%.

## 4 Discussion

PCV2 is an important pathogen of porcine circovirus disease (PCVD), which is highly prevalent in swine herds and severely affects the global pig industry (7, 25). Since the discovery of PCV3 in 2016, increasing evidence suggests that this strain poses a severe threat to both sows and piglets (26–28), and the emergence of PCV4 in 2019 has further complicated porcine circovirus disease diagnosis (17, 23). The prevalence of PCV3 and PCV4 infections in pigs is increasing, although the pathogenicity of PCV3 and PCV4 in this area remains controversial (29). Several diagnosis methods have been developed for PCV, including immunohistochemistry, ELISA, RT-PCR, loop-mediated isothermal amplification (LAMP), and real-time RT-PCR. However, ELISA and immunohistochemistry are time-consuming and cannot detect viral DNA/cDNA copy numbers. Conversely, qPCR has been widely used for the diagnosis of related diseases because of its advantages over conventional PCR, such as high sensitivity and specificity for high-throughput screening and viral load quantification using small volumes. Based on the above objectives, we developed a multiplex qPCR assay for the simultaneous differential diagnosis of PCV2, PCV3, and PCV4. Through sequence alignment of the complete circovirus genome, primers and probes were finally designed for the rep gene of PCV2, the cap gene of PCV3 and PCV4. After system optimization, the primers and probes have high specificity and are not affected by other binding sites. The assay can detect PCVs plasmid at a concentration of 3.8 copies/μL, which is more sensitive than the assay established by Chen and Zou et al. (30, 31). Additionally, the coefficients of variation (CV) of the intra- and inter-group for this method ranged from 0.10 to 0.77% and 0.47 to 1.45%, providing good sensitivity and reproducibility. Thus, the newly established assay provides a relevant tool for more rapid and accurate diagnosis of different species of PCVs.

PCVs distribution and spread are quite prevalent among pig populations in China. According to Li et al., 15,130 samples from eight provinces were tested for PCV2 from 2018 to 2021, and overall PCV2 positivity rate was 24.04% (3,637/15,130) in some regions, with Guangdong province testing positive at a rate of 17.18% (315/1834) (32). Additionally, Nan et al. tested 573 samples for PCV2 from 132 pig farms in northern Guangdong Province from 2016 to 2021 and found a positive rate of 51.38% (297/573) (33). Recently, Zou et al. established a multiplex qPCR assay and tested 535 samples from Shandong, Zhejiang, Jiangsu and Anhui provinces in eastern China from 2020 to 2022. PCV2, PCV3 and PCV4 infection rates were 31.03, 30.09 and 30.84%, respectively, and the mixed infection rate was 28.22% (31). The above data suggests that PCV2 and PCV3 are more widely distributed and transmitted in swine herds in some regions of China. In this study, we used a newly established qPCR assay on porcine blood and nasopharyngeal swab samples collected in Guangdong Province in 2020–2021, and found that the infection rates of PCV2 and PCV3 were relatively high (65.18 and 29.27%, respectively), and PCV2/PCV3 co-infection rate of 23.17%, which was higher than that recently reported for circoviruses in Guangdong Province (34). The differences in these data can be attributed to various factors, including sample types, sample sizes, geographic location, health status, immune response, PCR detection methods, and other variables. Overall, the results of this study indicated that PCV2 and PCV3 are more widely distributed and spread in pig farms in Guangdong Province, but PCV4 was not detected in any of the clinical samples.

Since the first reported case of PCV2 infection in China in 2000, PCV2a was the predominant epidemic subtype in Chinese pig herds (35), but around 2003, PCV2b replaced PCV2a as the new major epidemic subtype (36). In recent years, PCV2d has become an emerging major prevalent genotype in China (37). In our study, 17 PCV2 strains were isolated with 96.7 to 100% complete genome sequence identity and 94.0 to 100% ORF2 identity, 6 of which belonged to PCV2b and 11 to PCV2d. Four PCV3 strains had 98.9 to 99.4% complete genome sequence identity and 98.3 to 99.2% ORF2 identity, of which 2 strains belonged to PCV3b and 2 belonged to PCV3a-1. Unfortunately, due to limitations of certain conditions, the complete genome of all the samples was not obtained so this data may not fully represent all Guangdong Province PCVs genotypes. Curiously, we did not obtain PCV2a sequence, which seems to be inconsistent with previous reports (33, 34). However, our findings indicating that PCV2d is still the main prevalent genotype, which is consistent with previous reports (38). The first case of PCV4 transmission was detected in Hunan Province in 2019, and recent studies have detected PCV4 epidemics in Henan, Jiangsu, and Shanxi provinces (5, 23). However, we did not detect PCV4 in swine samples from Guangdong Province, which may be due to the short time for sample collection, insufficient sample number and sample type. To determine the prevalence of PCV4, continuous monitoring of pigs from Guangdong Province is needed. Moreover, due to changing PCV2 subtypes and the presence of PCV3 and PCV4, the current commercial PCV2 vaccine has a relatively singular scope of action, and co-infections of PCVs with other porcine reproductive disorders have become common (39, 40). Therefore, it is necessary to conduct regular testing on swine farms to grasp the status of circovirus infection in a timely manner, in order to provide a basis for the prevention and control PCV.

## 5 Conclusion

We successfully established a triple TaqMan real-time PCR assay for simultaneous PCV2, PCV3 and PCV4 detection. The method exhibits excellent specificity, sensitivity and reproducibility. Additionally, molecular epidemiologic testing of PCVs conducted in Guangdong Province in 2020 to 2021 showed that the PCV2 infection rate was 65.18% (481/738), PCV3 infection rate was 29.27% (216/738), and PCV2/PCV3 co-infection rate was 23.17% (171/738). Finally, 17 PCV2 and 4 PCV3 complete genome sequences were obtained in this study. These results indicate that PCV2 and PCV3 are prevalent in swine herds in Guangdong Province, China, and that co-infections of these two viruses are common. The above data provide an important reference for PCVs epidemiological investigation and genetic variation in Guangdong Province, China.

## Data availability statement

The original contributions presented in the study are publicly available. This data can be found here: https://www.ncbi.nlm.nih.gov/nuccore/; MZ667312, MZ667314-MZ667329, MZ667331-MZ667334.

## Ethics statement

The animal studies were approved by Institute of Animal Health, Guangdong Academy of Agricultural Sciences. The studies were conducted in accordance with the local legislation and institutional requirements. Written informed consent was obtained from the owners for the participation of their animals in this study.

## Author contributions

PZ: Formal analysis, Writing – original draft, Data curation. ZR: Methodology, Writing – original draft. XG: Formal analysis, Methodology, Writing – original draft. MZ: Data curation, Writing – original draft. YW: Methodology, Writing – original draft, Software. JC: Data curation, Writing – original draft. GW: Data curation, Writing – review & editing. HX: Data curation, Writing – original draft. RC: Data curation, Writing – original draft. SL: Methodology, Writing – original draft. XW: Conceptualization, Methodology, Project administration, Writing – review & editing.
