# Supplementary material for: Development and application of a TaqMan-probe-based multiplex real-time PCR assay for simultaneous detection of porcine circovirus 2, 3, and 4 in Guangdong province of China
Source: Front Vet Sci. 2024 Apr 26;11:1353439. doi: 10.3389/fvets.2024.1353439 (PMC11085253; doi:10.3389/fvets.2024.1353439)
Supplement: Supplementary file 1 [file Data_Sheet_1.docx]

Supplementary Material

**Supplementary Table 1** Complete genome sequence obtained in this study.

| **Number** | **Genotype** | **GenBank** | **Province/City** | **Year** |
| --- | --- | --- | --- | --- |
| 1 | PCV2d | MZ667312 | JiangMen | 2018 |
| 2 | PCV2d | MZ667314 | YangChun | 2017 |
| 3 | PCV2d | MZ667315 | YangChun | 2017 |
| 4 | PCV2d | MZ667316 | JiangMen | 2017 |
| 5 | PCV2b | MZ667317 | YangChun | 2017 |
| 6 | PCV2b | MZ667318 | JiangMen | 2016 |
| 7 | PCV2b | MZ667319 | MaoMing | 2016 |
| 8 | PCV2b | MZ667320 | JiangMen | 2015 |
| 9 | PCV2b | MZ667321 | JiangMen | 2017 |
| 10 | PCV2d | MZ667322 | Guangdong | 2016 |
| 11 | PCV2d | MZ667323 | Guangdong | 2015 |
| 12 | PCV2d | MZ667324 | YangJiang | 2017 |
| 13 | PCV2b | MZ667325 | HuiZhou | 2015 |
| 14 | PCV2d | MZ667326 | GuangZhou | 2021 |
| 15 | PCV2d | MZ667327 | YangJiang | 2021 |
| 16 | PCV2d | MZ667328 | FoShan | 2021 |
| 17 | PCV2d | MZ667329 | GuangDong | 2021 |
| 18 | PCV3a-1 | MZ667331 | MaoMing | 2016 |
| 19 | PCV3b | MZ667332 | GuangDong | 2021 |
| 20 | PCV3b | MZ667333 | GuangDong | 2021 |
| 21 | PCV3a-1 | MZ667334 | GuangDong | 2021 |

**Supplementary Table 2** Optimization of PCV2 primer concentrations of multiplex qPCR assay.

| **Primer concentration (nM)** | **template** | **Copy number (copies/μL)** | | | | | **amplification efficiency (%)** |
| --- | --- | --- | --- | --- | --- | --- | --- |
|  |  | **3.80×10^6^** | **3.80×10^5^** | **3.80×10^4^** | **3.80×10^3^** | **3.80×10^2^** |  |
| 100 | PCV2 | 18.01 | 22.59 | 26.66 | 29.04 | 33.37 | 85.83 |
|  | PCV3 | 17.14 | 21.53 | 25.62 | 28.23 | 31.30 | 93.00 |
|  | PCV4 | 15.74 | 20.57 | 24.92 | 27.48 | 31.69 | 80.99 |
| 150 | PCV2 | 17.44 | 21.37 | 25.19 | 28.21 | 29.92 | 106.28 |
|  | PCV3 | 17.66 | 21.41 | 24.86 | 27.87 | 29.80 | 111.50 |
|  | PCV4 | 15.08 | 19.40 | 23.18 | 25.60 | 27.47 | 110.28 |
| 200 | PCV2 | 17.56 | 21.65 | 26.09 | 27.71 | 30.06 | 109.87 |
|  | PCV3 | 17.26 | 21.47 | 25.69 | 27.15 | 29.88 | 110.58 |
|  | PCV4 | 14.75 | 18.74 | 22.60 | 24.53 | 26.85 | 115.44 |
| 250 | PCV2 | 17.86 | 21.31 | 26.20 | 28.24 | 30.61 | 103.40 |
|  | PCV3 | 16.90 | 20.80 | 25.24 | 27.32 | 29.70 | 104.80 |
|  | PCV4 | 16.07 | 20.05 | 24.48 | 26.56 | 29.37 | 100.46 |
| 300 | PCV2 | 17.77 | 21.88 | 26.45 | 28.51 | 31.21 | 98.80 |
|  | PCV3 | 17.45 | 23.13 | 25.51 | 27.81 | 31.06 | 105.82 |
|  | PCV4 | 15.42 | 19.02 | 23.48 | 25.24 | 28.68 | 102.04 |
| 350 | PCV2 | 17.32 | 21.48 | 25.02 | 27.95 | 31.22 | 95.79 |
|  | PCV3 | 17.23 | 21.33 | 24.51 | 27.44 | 30.31 | 104.12 |
|  | PCV4 | 15.32 | 19.84 | 23.22 | 26.04 | 29.53 | 94.47 |
| 400 | PCV2 | 17.23 | 20.36 | 24.00 | 26.69 | 31.21 | 95.72 |
|  | PCV3 | 15.90 | 19.04 | 22.91 | 25.58 | 30.01 | 93.95 |
|  | PCV4 | 15.48 | 18.17 | 21.81 | 24.17 | 28.81 | 102.39 |
| 450 | PCV2 | 17.40 | 21.52 | 25.97 | 27.81 | 29.81 | 109.62 |
|  | PCV3 | 17.25 | 21.20 | 24.00 | 27.35 | 28.85 | 119.14 |
|  | PCV4 | 15.29 | 19.28 | 24.45 | 26.17 | 28.23 | 101.91 |
| 500 | PCV2 | 17.57 | 21.66 | 26.69 | 28.51 | 33.53 | 81.11 |
|  | PCV3 | 16.58 | 20.32 | 25.33 | 27.30 | 32.01 | 83.77 |
|  | PCV4 | 15.78 | 19.24 | 24.63 | 26.58 | 30.94 | 84.30 |

**Supplementary Table 3** Optimization of PCV3 primer concentrations of multiplex qPCR assay.

| **Primer concentration (nM)** | **template** | **Copy number (copies/μL)** | | | | | **amplification efficiency (%)** |
| --- | --- | --- | --- | --- | --- | --- | --- |
|  |  | **3.80×10^6^** | **3.80×10^5^** | **3.80×10^4^** | **3.80×10^3^** | **3.80×10^2^** |  |
| 100 | PCV2 | 18.97 | 22.70 | 26.82 | 29.63 | 32.80 | 89.24 |
|  | PCV3 | 18.13 | 22.47 | 26.49 | 29.37 | 32.43 | 84.06 |
|  | PCV4 | 17.19 | 21.88 | 26.06 | 29.33 | 31.68 | 76.32 |
| 150 | PCV2 | 20.71 | 24.32 | 27.43 | 30.52 | 33.12 | 102.91 |
|  | PCV3 | 20.24 | 23.91 | 26.62 | 29.60 | 31.24 | 111.24 |
|  | PCV4 | 20.20 | 23.74 | 26.42 | 30.37 | 32.47 | 100.12 |
| 200 | PCV2 | 15.85 | 19.80 | 23.05 | 26.10 | 29.73 | 96.84 |
|  | PCV3 | 14.68 | 19.17 | 22.45 | 25.76 | 29.02 | 87.85 |
|  | PCV4 | 14.71 | 18.31 | 21.23 | 24.25 | 28.25 | 107.52 |
| 250 | PCV2 | 18.54 | 22.97 | 26.51 | 29.52 | 33.30 | 87.98 |
|  | PCV3 | 18.32 | 22.69 | 25.84 | 29.10 | 32.42 | 91.32 |
|  | PCV4 | 15.73 | 19.97 | 23.71 | 27.80 | 31.83 | 77.96 |
| 300 | PCV2 | 15.58 | 19.69 | 23.11 | 25.99 | 29.02 | 94.36 |
|  | PCV3 | 15.13 | 19.20 | 22.73 | 25.56 | 28.28 | 93.73 |
|  | PCV4 | 14.39 | 17.81 | 21.42 | 24.37 | 26.36 | 98.64 |
| 350 | PCV2 | 19.61 | 23.71 | 26.89 | 29.69 | 31.52 | 99.17 |
|  | PCV3 | 20.10 | 23.41 | 27.41 | 29.97 | 30.35 | 98.39 |
|  | PCV4 | 18.72 | 22.90 | 26.10 | 28.52 | 32.21 | 102.65 |
| 400 | PCV2 | 21.59 | 23.94 | 27.98 | 30.59 | 31.71 | 109.97 |
|  | PCV3 | 15.29 | 18.73 | 22.31 | 25.49 | 28.16 | 96.14 |
|  | PCV4 | 18.85 | 22.64 | 27.41 | 30.38 | 32.49 | 79.50 |
| 450 | PCV2 | 21.69 | 25.84 | 28.94 | 32.10 | 35.94 | 93.95 |
|  | PCV3 | 20.09 | 23.79 | 26.56 | 29.79 | 31.82 | 105.96 |
|  | PCV4 | 21.42 | 25.53 | 28.54 | 31.84 | 35.27 | 96.80 |
| 500 | PCV2 | 15.42 | 19.49 | 23.26 | 26.50 | 29.35 | 86.29 |
|  | PCV3 | 19.38 | 23.48 | 26.41 | 28.97 | 30.17 | 106.76 |
|  | PCV4 | 14.29 | 17.14 | 21.51 | 24.89 | 27.09 | 89.00 |

**Supplementary Table 4** Optimization of PCV4 primer concentrations of multiplex qPCR assay.

| **Primer concentration (nM)** | **template** | **Copy number (copies/μL)** | | | | | **amplification efficiency (%)** |
| --- | --- | --- | --- | --- | --- | --- | --- |
|  |  | **3.80×10^6^** | **3.80×10^5^** | **3.80×10^4^** | **3.80×10^3^** | **3.80×10^2^** |  |
| 100 | PCV2 | 18.79 | 22.28 | 25.74 | 29.20 | 32.19 | 97.95 |
|  | PCV3 | 18.22 | 21.80 | 25.15 | 28.41 | 31.47 | 100.46 |
|  | PCV4 | 18.80 | 21.65 | 25.33 | 29.44 | 31.98 | 96.26 |
| 150 | PCV2 | 17.69 | 20.99 | 24.05 | 28.04 | 31.12 | 97.20 |
|  | PCV3 | 17.18 | 20.22 | 23.33 | 27.24 | 30.36 | 99.33 |
|  | PCV4 | 17.28 | 20.38 | 23.49 | 27.63 | 30.97 | 94.43 |
| 200 | PCV2 | 15.70 | 19.23 | 22.77 | 26.35 | 29.11 | 97.08 |
|  | PCV3 | 15.39 | 18.73 | 22.19 | 25.76 | 28.38 | 100.88 |
|  | PCV4 | 14.31 | 16.65 | 20.49 | 23.97 | 26.90 | 103.09 |
| 250 | PCV2 | 19.98 | 23.46 | 26.80 | 30.61 | 33.53 | 95.87 |
|  | PCV3 | 19.47 | 23.04 | 26.57 | 30.60 | 33.19 | 93.07 |
|  | PCV4 | 17.81 | 21.46 | 25.42 | 30.12 | 32.37 | 83.95 |
| 300 | PCV2 | 17.36 | 20.91 | 24.37 | 27.87 | 30.66 | 98.60 |
|  | PCV3 | 17.03 | 20.43 | 23.92 | 27.34 | 29.60 | 105.12 |
|  | PCV4 | 15.34 | 18.89 | 22.57 | 26.56 | 29.11 | 92.31 |
| 350 | PCV2 | 16.15 | 18.75 | 23.13 | 26.84 | 30.09 | 89.67 |
|  | PCV3 | 15.39 | 17.97 | 22.27 | 25.81 | 28.75 | 94.69 |
|  | PCV4 | 13.95 | 17.61 | 21.33 | 24.80 | 28.46 | 88.87 |
| 400 | PCV2 | 15.81 | 19.23 | 23.08 | 26.50 | 29.58 | 93.76 |
|  | PCV3 | 15.57 | 18.81 | 22.60 | 25.98 | 29.04 | 96.41 |
|  | PCV4 | 13.96 | 17.26 | 21.51 | 24.69 | 28.01 | 91.18 |
| 450 | PCV2 | 15.87 | 19.51 | 23.14 | 26.84 | 29.91 | 91.60 |
|  | PCV3 | 15.40 | 18.88 | 22.38 | 25.94 | 29.04 | 95.53 |
|  | PCV4 | 13.99 | 18.01 | 21.33 | 25.03 | 28.04 | 92.64 |
| 500 | PCV2 | 17.97 | 20.98 | 25.13 | 27.75 | 31.80 | 98.88 |
|  | PCV3 | 16.92 | 20.13 | 24.07 | 26.58 | 30.90 | 101.26 |
|  | PCV4 | 15.69 | 19.32 | 23.56 | 26.01 | 31.10 | 92.35 |

**Supplementary Table 5** Optimization of probe concentrations of multiplex qPCR assay.

| PCV2 (FAM) | | | | | | | |
| --- | --- | --- | --- | --- | --- | --- | --- |
| **Probe concentration (nM)** | **template** | **Copy number (copies/μL)** | | | | | **amplification efficiency (%)** |
|  |  | **3.80×10^6^** | **3.80×10^5^** | **3.80×10^4^** | **3.80×10^3^** | **3.80×10^2^** |  |
| 50 | PCV2 | 17.15 | 20.36 | 24.34 | 26.67 | 31.11 | 95.95 |
|  | PCV3 | 16.70 | 19.94 | 24.00 | 26.35 | 30.77 | 94.73 |
|  | PCV4 | 15.39 | 18.95 | 23.97 | 24.93 | 29.16 | 98.76 |
| 100 | PCV2 | 16.28 | 19.82 | 23.12 | 26.15 | 29.71 | 100.12 |
|  | PCV3 | 16.04 | 19.67 | 22.88 | 26.06 | 29.73 | 97.75 |
|  | PCV4 | 15.00 | 18.04 | 21.90 | 24.60 | 30.67 | 102.39 |
| 150 | PCV2 | 17.21 | 20.33 | 24.31 | 27.00 | 31.33 | 98.88 |
|  | PCV3 | 16.45 | 19.39 | 23.25 | 25.89 | 30.37 | 104.53 |
|  | PCV4 | 13.28 | 16.49 | 19.54 | 22.74 | 28.22 | 108.05 |
| 200 | PCV2 | 18.00 | 21.99 | 27.12 | 28.14 | 30.92 | 105.40 |
|  | PCV3 | 18.02 | 22.14 | 27.12 | 28.07 | 30.63 | 109.42 |
|  | PCV4 | 14.56 | 17.15 | 20.94 | 23.22 | 29.19 | 115.44 |
| PCV3 (HEX**)** | | | | | | | |
| **Probe concentration (nM)** | **template** | **Copy number** (**copies/μL)** | | | | | **amplification efficiency (%)** |
|  |  | **3.80×10^6^** | **3.80×10^5^** | **3.80×10^4^** | **3.80×10^3^** | **3.80×10^2^** |  |
| 50 | PCV2 | 16.42 | 19.26 | 22.91 | 26.35 | 29.90 | 96.65 |
|  | PCV3 | 15.82 | 18.50 | 22.82 | 26.21 | 29.63 | 91.89 |
|  | PCV4 | 13.94 | 16.38 | 20.35 | 23.49 | 27.01 | 99.87 |
| 100 | PCV2 | 15.84 | 18.58 | 22.16 | 27.48 | 29.06 | 99.04 |
|  | PCV3 | 15.29 | 18.25 | 21.84 | 27.14 | 28.34 | 101.39 |
|  | PCV4 | 13.87 | 16.50 | 20.04 | 25.01 | 26.86 | 101.30 |
| 150 | PCV2 | 16.04 | 19.32 | 23.26 | 27.87 | 30.21 | 86.67 |
|  | PCV3 | 15.43 | 18.37 | 22.58 | 27.32 | 29.59 | 85.49 |
|  | PCV4 | 13.79 | 16.41 | 21.07 | 24.78 | 27.04 | 93.54 |
| 200 | PCV2 | 17.02 | 20.59 | 24.24 | 27.79 | 30.96 | 92.78 |
|  | PCV3 | 16.57 | 19.61 | 22.77 | 26.13 | 29.47 | 103.89 |
|  | PCV4 | 14.62 | 17.87 | 21.17 | 24.66 | 28.35 | 95.87 |
| PCV4 (CY5**)** | | | | | | | |
| **Probe concentration (nM)** | **template** | **Copy number** (**copies/μL)** | | | | | **amplification efficiency (%)** |
|  |  | **3.80×10^6^** | **3.80×10^5^** | **3.80×10^4^** | **3.80×10^3^** | **3.80×10^2^** |  |
| 50 | PCV2 | 15.46 | 19.11 | 22.94 | 26.20 | 29.40 | 93.18 |
|  | PCV3 | 15.35 | 18.78 | 22.58 | 25.64 | 29.03 | 95.99 |
|  | PCV4 | 14.00 | 17.78 | 21.55 | 24.57 | 28.05 | 93.47 |
| 100 | PCV2 | 16.11 | 19.44 | 22.89 | 26.53 | 29.48 | 97.51 |
|  | PCV3 | 15.51 | 19.00 | 22.25 | 25.81 | 28.76 | 99.62 |
|  | PCV4 | 14.57 | 18.08 | 21.04 | 24.77 | 27.95 | 99.05 |
| 150 | PCV2 | 16.16 | 19.58 | 23.05 | 26.57 | 29.65 | 96.96 |
|  | PCV3 | 15.60 | 18.77 | 22.41 | 25.75 | 28.83 | 99.09 |
|  | PCV4 | 14.61 | 17.82 | 20.94 | 24.20 | 27.28 | 106.66 |
| 200 | PCV2 | 16.82 | 19.98 | 23.70 | 27.37 | 30.96 | 90.70 |
|  | PCV3 | 15.66 | 19.07 | 22.77 | 26.38 | 29.79 | 91.05 |
|  | PCV4 | 13.89 | 17.78 | 21.26 | 24.57 | 27.97 | 93.25 |
